# Commentary: Impact of Fecal Calprotectin Measurement on Decision-Making in Children with Inflammatory Bowel Disease

**DOI:** 10.3389/fped.2017.00133

**Published:** 2017-06-06

**Authors:** Andrew S. Day, Mustafa Adamji

**Affiliations:** ^1^Department of Paediatrics, Christchurch Hospital, Christchurch, New Zealand; ^2^Department of Paediatrics, University of Otago, Christchurch, New Zealand

**Keywords:** children, inflammatory bowel disease, fecal markers, calprotectin, Crohn disease

In their recent publication, El-Matary et al. (1) describe the utility of measurement of fecal calprotectin (FC) in the ongoing management of children with known inflammatory bowel disease (IBD). In this cohort of 77 children, FC was measured upon presentation with key changes in symptoms (most commonly abdominal pain and hematochezia). The child’s management was then adjusted according to the level of the FC, with a cutoff of 250 µg/g stool. Almost 90% of those with elevated FC had a change in their management, which resulted in a reduction in clinical activity indices over the subsequent 3–6 months. Repeated FC measurements were not available. Conversely, the majority of those with low FC (below the cutoff) had no change in management in the following months. Reassuringly, 94% of these children were judged to be in remission at their follow-up visit.

Non-invasive markers, especially those measured in stool, have become increasingly important and relevant in recent years in the management of IBD. Numerous markers have now been described. Although calprotectin has been the most utilized to date, other promising markers include S100A12, osteoprotegerin, lactoferrin, and M2 pyruvate kinase (2–4).

Generally, markers such as calprotectin provide greater specificity and sensitivity for the presence of gut inflammation than standard serum-based markers. In one assessment of routine serum markers at the time of diagnosis, erythrocyte sedimentation rate, C-reactive protein, albumin, and platelet counts were each normal in 19 (13%) and all abnormal in just 52 (36%) of 146 children with CD (5). Overall, the platelet count was most often abnormal in this group. Both FC and fecal S100A12 provided much greater utility than any of the same four serum markers in an earlier cohort of 31 children with IBD (6).

Although fecal markers have particular roles in the investigation of an individual with undifferentiated symptoms (to assess the need for further investigations and to reach a diagnosis), they also clearly have important roles in individuals diagnosed with IBD. Such roles include the monitoring of disease control, assessment of response to an intervention, assessment of mucosal healing (MH), and to provide an assessment of the risk of relapse in the coming months.

The current report focused upon children with a change in symptoms, such as the development of rectal bleeding. The key issues in this context are to ascertain whether the change in symptoms is related to an exacerbation of disease, or due to other factors such as an intercurrent infection or due to functional overlap. Both enteric infections and irritable bowel syndrome can result in an elevated FC. Prompt access to FC measurement clearly provides guidance as to the clinical management required. In practice, however, one would otherwise consider the pattern of symptoms, the routine blood tests, and examination findings and anthropometry as well. It would seem reasonable and appropriate to consider FC measurement in addition to these assessments, rather than instead.

There are some data suggesting that FC levels may vary according to disease location, with it being less reliable in small bowel inflammation (7). Assessment at baseline, at the time of initial diagnostic endoscopy, along with serial measurements over time may be of assistance for the individual patient. Accordingly, a change in FC from a prior measurement may be more helpful than a one-off level.

The other potential roles of fecal markers in monitoring disease progress are also important, especially in children. Several reports indicate that the serial measurement of S100A12 or FC in individuals in clinical remission may predict the risk of a future relapse (8, 9). In addition, fecal markers may enable the early prediction of recurrence after ileo-colonic resection (10). The role of FC in predicting the achievement of MH is less clear: the data are not yet conclusive as to the correlation between FC and MH (11). In addition, FC may have a role in guiding the appropriate indication for repeat endoscopic assessment in children with established disease: optimizing the timing of endoscopy in children will clearly be of benefit to health administrations (given the cost of endoscopy) and also for children and their parents (given the inconvenience of endoscopy for children).

Clearly, the potential roles for FC and other non-invasive markers are likely to expand. Further understanding of the differential information provided by the various markers may lead to the use of more than one marker, or indeed to the use of a panel of markers.

In conclusion, the work of El-Matary and colleagues (1) provides further important information of the utility of FC in children with IBD. Additional prospective assessments are required, ideally with comparisons between available markers and with serial estimations over time.

## Author Contributions

AD formulated the plan for this commentary along with initial draft manuscript. MA reviewed the draft manuscript and assisted in revisions of the drafts of the manuscript.

## Conflict of Interest Statement

The authors declare that the research was conducted in the absence of any commercial or financial relationships that could be construed as a potential conflict of interest.
